# Locoregional Recurrence Risk in Breast Cancer Patients with Estrogen Receptor Positive Tumors and Residual Nodal Disease following Neoadjuvant Chemotherapy and Mastectomy without Radiation Therapy

**DOI:** 10.1155/2015/147476

**Published:** 2015-07-21

**Authors:** Shravan Kandula, Jeffrey M. Switchenko, Saul Harari, Carolina Fasola, Donna Mister, David S. Yu, Amelia B. Zelnak, Mylin A. Torres

**Affiliations:** ^1^Department of Radiation Oncology, Emory University School of Medicine, Atlanta, GA 30322, USA; ^2^Winship Cancer Institute, Emory University, Atlanta, GA 30322, USA; ^3^Department of Biostatistics and Bioinformatics, Emory University, Atlanta, GA 30322, USA; ^4^Department of Pathology, Emory University, Atlanta, GA 30322, USA; ^5^Department of Radiation Oncology, Stanford Medical Center, Stanford, CA 94305, USA; ^6^Department of Hematology and Medical Oncology, Emory University School of Medicine, Atlanta, GA 30322, USA

## Abstract

Among breast cancer patients treated with neoadjuvant chemotherapy (NAC) and mastectomy, locoregional recurrence (LRR) rates are unclear in women with ER+ tumors treated with adjuvant endocrine therapy without postmastectomy radiation (PMRT). To determine if PMRT is needed in these patients, we compared LRR rates of patients with ER+ tumors (treated with adjuvant endocrine therapy) with women who have non-ER+ tumors. 85 consecutive breast cancer patients (87 breast tumors) treated with NAC and mastectomy without PMRT were reviewed. Patients were divided by residual nodal disease (ypN) status (ypN+ versus ypN0) and then stratified by receptor subtype. Among ypN+ patients (*n* = 35), five-year LRR risk in patients with ER+, Her2+, and triple negative tumors was 5%, 33%, and 37%, respectively (*p* = 0.02). Among ypN+/ER+ patients, lymphovascular invasion and grade three disease increased the five-year LRR risk to 13% and 11%, respectively. Among ypN0 patients (*n* = 52), five-year LRR risk in patients with ER+, Her2+, and triple negative tumors was 7%, 22%, and 6%, respectively (*p* = 0.71). In women with ER+ tumors and residual nodal disease, endocrine therapy may be sufficient adjuvant treatment, except in patients with lymphovascular invasion or grade three tumors where PMRT may still be indicated.

## 1. Introduction

Traditionally, postmastectomy radiation (PMRT) decisions have been guided by pathologic findings in breast cancer patients treated with initial surgery. In this setting, data from several studies have led to guidelines which have identified patients most likely to benefit from PMRT: those with primary tumors greater than five centimeters, four or more positive lymph nodes (pN2), or one to three positive lymph nodes (pN1) with high-risk features such as extracapsular extension (ECE) and lymphovascular invasion (LVI) [1–3]. However, these same recommendations do not necessarily apply to patients treated with neoadjuvant chemotherapy (NAC) where the initial extent of disease is unknown and can be modified in as many as 80% of patients [4].

There are no published randomized trials to guide the use of PMRT in women treated with NAC [5]. Retrospective studies have suggested that both advanced initial clinical stage and residual pathologic nodal disease (ypN) are associated with a higher risk of locoregional recurrence (LRR) in women treated with NAC [6–11]. However, there are many instances in which the initial clinical stage is unclear despite physical exam and modern imaging. The inaccuracies of physical exam are best demonstrated by the results of the National Surgical Adjuvant Breast and Bowel Project (NSABP) B-04 which found that 40% of clinically node negative (cN0) patients on physical exam were actually pathologically node positive (pN+), while 25% of cN+ patients were actually pN0 [12]. Modern imaging has resulted in only modest improvements in detection of axillary nodal metastases, with broad sensitivity and specificity ranges reported for ultrasound (43.5–86.2% and 40.5–86.6%, resp.) [13–16], magnetic resonance imaging (MRI) (36–78% and 78–100%, resp.) [16–20], and full body fluorodeoxyglucose- (FDG-) positron emission tomography (PET)/computed tomography (CT) (20–100% and 75–100%, resp.) [21]. Therefore, clinical staging may not accurately reflect the extent of disease prior to NAC and may lead to under- or overtreatment with PMRT. Furthermore, other studies have indicated that residual nodal response following NAC plays a larger role in determining LRR risk than initial clinical stage or primary breast tumor response (ypT status) [9, 22]. Patients with complete nodal response to NAC were found to have a very low risk of LRR despite having locally advanced disease initially at presentation [23, 24]. Therefore, ypN status is arguably a more robust and consistent predictor of LRR in the NAC setting.

Nevertheless, as there is heterogeneity in the risk of LRR among pN1 patients, there is also potentially a spectrum of LRR risk among ypN1 patients. Few studies have examined the impact of receptor status on LRR risk in ypN+ or ypN0 patients. The LRR risk is unclear in patients with estrogen receptor positive (ER+) tumors and ypN+ disease who are treated with adjuvant endocrine therapy without PMRT. The Early Breast Cancer Trialists Collaborative Group meta-analysis demonstrated that the addition of PMRT significantly improved 15-year breast cancer-specific survival in patients with a greater than 10% LRR risk [25]. The Athena Breast Health Network thus adopted an absolute LRR risk threshold of 10% before recommending PMRT in patients treated with NAC [26]. The aim of our research was to compare LRR risk among breast cancer patients with ER+ tumors (treated with adjuvant endocrine therapy) and those patients with non-ER+ tumors following NAC and mastectomy without PMRT. Given the shortcomings of initial clinical staging, we also sought to identify additional objective pathological factors that contribute to a five-year LRR risk of greater than 10%.

## 2. Materials and Methods 

### 2.1. Patient Population

At our institution, NAC is typically administered in patients with large primary tumor to breast size ratios, locally advanced or initially unresectable breast cancers, and/or triple negative and Her2+ tumors. Following approval from the institutional review board, the medical records of breast cancer patients treated with modern anthracycline and/or taxane-based NAC between 1997 and 2011 were reviewed. 553 breast cancer patients (with noninflammatory, nonmetastatic cancer) were identified. After excluding patients who underwent mastectomy and PMRT (*n* = 295) or those who underwent breast-conservation therapy (lumpectomy and radiation therapy) (*n* = 173), a total of 85 patients (87 breast tumors) who underwent NAC, mastectomy, and lymph node evaluation without PMRT were identified. Receptor status was determined from immunohistochemistry (IHC) testing of the biopsy specimen. Fluorescence in situ hybridization (FISH) testing was typically performed in cases of 2+ Her2-positivity. Tumors were considered Her2-positive(+) with either 3+overexpression on IHC or gene amplification on FISH.

### 2.2. Treatments

Seventy-nine percent of patients received anthracycline-based chemotherapy; 64% of these patients received both an anthracycline and taxane. The remaining patients (21%) received taxane-based chemotherapy with the most common regimen consisting of docetaxel and cyclophosphamide for four cycles. Among patients with Her2+ breast cancers, 43% received trastuzumab.

Following NAC, all patients underwent mastectomy, in which 66% underwent modified radical mastectomy, 21% underwent simple mastectomy with sentinel lymph node biopsy alone, and 13% underwent nipple-sparing mastectomy with sentinel lymph node biopsy alone. The median number of lymph nodes dissected in patients who underwent axillary dissection and sentinel lymph node evaluation alone was 12 (range: 1–38) and 3 (range: 1–10), respectively. Following surgery, 10% of patients received adjuvant chemotherapy. Adjuvant endocrine therapy was received by all estrogen receptor positive (ER+) patients, consisting of tamoxifen alone, an aromatase inhibitor alone, or both (i.e., tamoxifen followed by an aromatase inhibitor) in 61%, 20%, and 19% of patients, respectively.

### 2.3. Data Collection and Statistical Analysis

Patients were divided by ypN status (ypN+ versus ypN0) and then stratified by receptor subtype (ER+/Her2− versus Her2+ versus triple negative). Pathologic factors, including biopsy grade within the primary, presence of LVI in the surgical specimen, and lymph node ratio (defined as the total number of positive lymph nodes divided by the total number of dissected lymph nodes in ypN+ patients), were analyzed in the various ypN patient and receptor subgroups to determine their additional impact on LRR risk. Institutional breast pathologists evaluated the biopsy specimen as well as the surgical specimens following neoadjuvant chemotherapy. LVI was assessed in the peritumoral tissue on hematoxylin and eosin-stained sections and identified as carcinoma cells present within an endothelial-lined lymphatic space or blood vessel (confirmation via a combined keratin/D240 cocktail assay). Biopsy grade was based on a three-tiered system which considered mitotic activity, tubule/gland formation, and nuclear pleomorphism. Numbers were allocated to various features and then totaled to assign the grade (3–5 equal to grade 1, 6-7 equal to grade 2, and 8-9 equal to grade 3). The presence of ECE and close or positive margins following mastectomy were not evaluated for their relationship with LRR due to the small numbers in our cohort (see Table 1).

LRR was defined as tumor recurrence in the ipsilateral chest wall or regional lymph nodes (axilla, internal mammary, supraclavicular, or infraclavicular fossa) at any time point (with or without distant metastases). Time to LRR and time to follow-up were calculated from date of diagnosis. Actuarial rates of LRR were calculated using the Kaplan-Meier method. The log-rank test was performed to evaluate impact of receptor class on LRR, with statistical significance defined as a *p* value of ≤0.05. Statistical analyses were performed using SPSS software (IBM SPSS version 19.0, Chicago, IL) and SAS software (SAS 9.3, Cary, NC).

## 3. Results

Patient and tumor characteristics are detailed in Table 1. The median age at diagnosis was 48 years (range: 30–87 years). Following NAC and mastectomy, 35 (40%) and 52 patients (60%) had positive and negative nodes, respectively. Among all patients, receptor status was ER+/Her2−, Her2+, and triple negative in 63%, 26%, and 26%, respectively. Median follow-up period was 52.6 months (range: 5.4–201.0 months). The initial clinical stage of the patients is presented in Table 2. Patients with clinical stage III breast cancers had significantly poorer five-year LRR than patients with stage I-II breast cancers (34% versus 8%, *p* < 0.01), but none of these patients were hormone receptor positive. Although advanced clinical stage/tumor size has previously been associated with LRR, there are clinical scenarios in which the initial clinical stage may be unclear [9, 10]. The focus of this study was to determine if objective pathological factors could unequivocally guide clinicians on LRR risk, particularly in patients with hormone receptor positive tumors treated with adjuvant hormone therapy.

### 3.1. ypN+ Patients, Receptor Status, and LRR

Among 35 ypN+ patients, 34 (97%) underwent an axillary dissection. Twenty-eight (80%) had fewer than four lymph nodes positive (ypN1mi or ypN1). The lymph node ratio was less than 20% in 66% of ypN+ patients. Among ypN+ patients, there was a statistically significant difference in five-year LRR risk across receptor subgroups (*p* = 0.02), with Her2+ and triple negative status associated with a considerably higher LRR risk (33% [*n* = 5] and 37% [*n* = 6], resp.) than ER+ status (5% [*n* = 24]). However, among ypN+/ER+ patients, the presence of LVI and grade three disease increased a patient's five-year LRR risk to 13% and 11%, respectively (see Table 3). Lymph node ratio was not associated with an increased LRR risk in these patients. Among ypN+ patients, the five-year rates of chest wall-only recurrences were 5%, 23%, and 25% in ER+, Her2+, and triple negative tumor subgroups, respectively, while five-year regional nodal-only recurrence rates were 0%, 0%, and 28%, respectively.

### 3.2. ypN0 Patients, Receptor Status, and LRR

Among ypN0 patients, the five-year LRR risk in patients with ER+, Her2+, and triple negative disease was 7% (*n* = 17), 22% (*n* = 18), and 6% (*n* = 17), respectively (*p* = 0.71). In ypN0/ER+ patients, the presence of LVI and grade three disease did not impact LRR risk. In patients with ypN0/triple negative breast cancer, the presence of grade 3 disease, however, increased the five-year LRR risk to 13%, but the presence of LVI did not increase LRR above 10% among these patients (see Table 4). Among ypN0 patients, the five-year rates of chest wall-only recurrences were 0%, 17%, and 0% for the ER+, Her2+, and triple negative tumor subgroups, respectively, while the five-year regional nodal-only recurrence rates were 7%, 22%, and 6%, respectively.

## 4. Discussion

Our findings indicate that receptor and ypN status may identify groups of patients in which PMRT may be omitted after NAC and mastectomy. Patients with ypN+/ER+ tumors had a significantly lower LRR risk than women with ypN+/triple negative or ypN+/Her2+ tumors. Based on a previously established LRR risk threshold of less than or equal to 10% [26, 27], our results suggest that ypN+/ER+ patients without LVI or grade 3 disease have a five-year LRR risk of 5% and may be sufficiently treated with adjuvant endocrine therapy and avoid PMRT. Among ypN0 patients, ER+ or triple negative (without high grade disease) status was also associated with a low five-year LRR risk (7% and 6%, resp.), although, in the ypN0/triple negative patients, grade 3 disease increased the five-year LRR risk to 13%.

While the significance of ypN stage has been previously illustrated [6–9, 11, 26, 28–30], the influence of receptor status on LRR risk in the NAC setting has not been fully investigated. A recent combined analysis of NSABP-18 and B-27 demonstrated that in patients treated with NAC and mastectomy, clinical tumor size and nodal status and pathological primary and nodal response (after NAC) were significant predictors of LRR [9]. Notably, rates of LRR were significantly above 10% for all subsets of patients with ypN1 disease. However, neither NSABP B-18 nor B-27 contained information on receptor status, and tamoxifen was administered based on patient age rather than receptor status. We chose to focus our analysis on objective factors easily ascertained from pathology reports in order to simplify clinical decisions regarding PMRT in NAC breast cancer patients, as there are many situations in which the initial clinical stage is not clear. By incorporating receptor status along with appropriate endocrine therapy, we were able to identify a patient cohort with ypN1 disease who may not carry a greater than 10% LRR risk.

Our results are consistent with the recently proposed low-risk group suggested by a breast cancer physician panel of the Athena Breast Health Network [26]. Based on available literature and clinical case scenarios, the authors applied the American College of Radiology (ACR) Appropriateness Criteria modified Delphi methodology (for establishing expert consensus) to identify patients treated with NAC for whom PMRT may be safely omitted. Their low-risk group (corresponding to a less than or equal to 10% LRR risk) included patients with ypN0 (including triple negative status) tumors and those with ypN1, ER+ disease, age greater than or equal to 35 years, and no presence of LVI or ECE. Tumor grade was not included in their analysis, but high grade disease on biopsy appeared to predict for a higher LRR risk in our patients with ypN+/ER+ and ypN0/triple negative status.

Compared to patients with ypN+/ER+ tumors, patients with ypN+ disease and triple negative or Her2+ tumors had a considerably higher LRR risk (greater than 30%) regardless of other pathological factors. These findings may be influenced by the inherent association of receptor status with tumor biology and more aggressive and advanced disease seen in women with Her2+ or triple negative disease at diagnosis (Table 2). As illustrated in a recent meta-analysis [31], patients with Her2+ and triple negative tumors are expected to achieve much higher pathological complete response rates compared to women with ER+ tumors. Thus, residual disease in patients with ER+ tumors may more accurately represent the initial disease extent. For this reason, ypN1/ER+ patients may forego PMRT in the absence of factors, such as LVI or high grade disease, which are associated with high LRR risk in pN1 patients treated with initial surgery [32, 33]. Residual nodal disease following NAC in patients with triple negative and Her2+ tumors is however concerning, as it may be indicative of even greater nodal burden prior to NAC and/or tumor resistance to systemic therapy. Therefore, when these patients do not develop a complete nodal response to NAC, they are at significant risk for LRR.

Another factor which may influence LRR risk is the size of the residual primary tumor (ypT stage), especially in relation to pathological complete response (pCR) (ypT0/isN0), which is highly predictive for recurrence-free survival [34]. The small percentage of pCRs (15%) in our cohort did not afford a meaningful analysis. Among the entire cohort, ypT stage was not a predictor of LRR, regardless of achievement of a pCR. Our results did suggest a trend in decreased 5-year LRR risk for residual primary tumor sizes of ≤2 cm (including ypT0, ypTis, and ypT1) compared with residual primary tumor sizes of >2 cm (ypT2 or greater) (10% versus 21%, *p* = 0.06). This result is consistent with prior literature [6]. Although ypT stage is an important factor, multiple studies have demonstrated that ypN status is a stronger predictor of LRR risk [9] and even overall survival [22].

Several strengths and limitations of this study warrant consideration. The majority of our ypN+/ER+ patients (92%) had less than four positive lymph nodes, and thus PMRT may still be needed in patients with ypN2 disease regardless of receptor status. Furthermore, only four subjects had positive nodes with ECE and 12 had close or positive margins following mastectomy. These small patient numbers precluded meaningful analysis with either of these pathologic factors, and therefore, our findings may not be broadly applied to patients with these tumor characteristics. Moreover, only 43% of the patients with Her2+ tumors in our study received trastuzumab, as they were treated prior to when this became standard of care. Among patients with Her2+ tumors in our study, the 5-year LRR risk for patients treated with and without trastuzumab was 12% (*n* = 10) and 32% (*n* = 13), respectively (*p* = 0.49). Although this difference was not statistically significant, our data and others suggest that trastuzumab may play an important role in LRR control [35]. However, even in patients treated with trastuzumab, the LRR rate was still above 10% and indicates that these patients may still derive benefit from PMRT. Lastly, our cohort of NAC patients treated with mastectomy and without PMRT is relatively large when compared with prior institutional series [McGuire et al. (*n* = 34) [10] and Nagar et al. (*n* = 43)] [30]. Furthermore, our study is unique in that all patients with ER+ tumors received endocrine therapy.

## 5. Conclusion

In conclusion, this study represents the first effort to examine the influence of receptor and nodal status on LRR risk among patients treated with NAC and mastectomy. Among patients with ER+ tumors and residual nodal disease, the risk of LRR is low and endocrine therapy appears to be sufficient adjuvant treatment, except in women with LVI or grade three tumors where PMRT may still be warranted. These observations corroborate and complement a recently proposed low-risk group of NAC breast cancer patients who may forego PMRT [26]. Conversely, PMRT appears warranted in women with ypN+ disease with Her2+ or triple negative tumors, who are at high risk of LRR regardless of other clinicopathologic factors. Thus, particularly in the setting of uncertain clinical stage, receptor and ypN status may help guide PMRT decisions. Our results must be validated in future, prospective studies.

## Figures and Tables

**Table 1:** Patient and tumor characteristics (*N* = 87).

Characteristic	*N* (%)
Age at diagnosis	
Median (range)	48 yrs (31–87 yrs)
Biopsy grade	
1	11 (13%)
2	27 (31%)
3	41 (47%)
Unknown	8 (9%)
Estrogen receptor status	
Positive	55 (63%)
Negative	32 (37%)
Progesterone receptor status	
Positive	40 (46%)
Negative	45 (52%)
Unknown	2 (2%)
Her2+ receptor status	
Positive	23 (26%)
Negative	59 (68%)
Unknown	5 (6%)
Triple negative receptor status	23 (26%)
ypT stage	
T0	7 (8%)
Tis	11 (13%)
T1mi/T1	44 (51%)
T2	20 (23%)
T3/T4	4 (4%)
Unknown	1 (1%)
ypN stage	
N0/0(i+)	52 (60%)
N+	35 (40%)
N1mi	3
N1a	25
N2a	6
N3a	1
LVI	
Yes	18 (21%)
No	67 (77%)
Unknown	2 (2%)
Close or positive margins	
Yes	12 (14%)
No	75 (86%)
Extranodal extension	
Yes	4 (5%)
No	82 (94%)
Unknown	1 (1%)
Lymph node ratio	
0%	52 (60%)
0–20%	23 (26%)
>20%	12 (14%)

yrs, years; LVI, lymphovascular invasion.

**Table 2:** Initial clinical stage of patients by receptor type.

Clinical stage	Tumor receptor type		
	ER+	Her2+	Triple negative
IA-IB	3	2	6
IIA	14	11	5
IIB	22	5	10
IIIA–IIIC	0	4	1
Unknown	2	1	1

**Table 3:** ypN/receptor groups associated with a >10% five-year LRR risk.

	5-year risk of LRR
ypN+	
Triple negative	37%^*∗*^
Her2+	33%^*∗*^
ER+, +LVI	13%
ER+, grade 3	11%

ypN0	
Her2+	22%^*∗*^
Triple negative, grade 3	13%

LRR, locoregional recurrence; LVI, lymphovascular space invasion.

*∗* denotes LRR risk without specification of other adverse pathological features.

**Table 4:** ypN/receptor groups associated with a ≤10% five-year LRR risk.

	5-year risk of LRR
ypN+	
ER+	5%^*∗*^

ypN0	
ER+	7%^*∗*^
Triple negative	6%^*∗*^

LRR, locoregional recurrence.

*∗* denotes LRR risk without specification of other adverse pathological features.
